# Prevalence, intensity and risk factors of tungiasis in Kilifi County, Kenya II: Results from a school-based observational study

**DOI:** 10.1371/journal.pntd.0007326

**Published:** 2019-05-16

**Authors:** Lynne Elson, Susanne Wiese, Hermann Feldmeier, Ulrike Fillinger

**Affiliations:** 1 KEMRI-Wellcome Trust Research Programme, Kilifi, Kenya; 2 Nuffield Department of Medicine, Oxford University, Oxford, United Kingdom; 3 Institute of Microbiology and Infection Immunology, Charité University Medicine, Berlin, Germany; 4 International Centre of Insect Physiology and Ecology, Human Health Theme, Thomas Odhiambo Campus, Mbita, Kenya; Saudi Ministry of Health, SAUDI ARABIA

## Abstract

**Introduction:**

Awareness of the public health importance of tungiasis has been growing in East Africa in recent years, but data on epidemiological characteristics necessary for the planning and implementation of control measures do not exist. The work presented here was part of a larger cross-sectional study on the epidemiology of tungiasis in coastal Kenya and aims at identifying risk factors of tungiasis and severe disease in school children.

**Methods:**

A total of 1,829 students of all age groups from five schools and 56 classes were clinically examined for tungiasis on their feet based on standardized procedures and observations made about the school infrastructure. To investigate the impact of school holidays, observations were repeated after school holidays in a subset of children in one school. In an embedded case-control study, structured interviews were conducted with 707 students in the five schools to investigate associations between tungiasis and household infrastructure, behaviour and socio-economic status.

**Results:**

The overall prevalence of tungiasis was 48%; children below the age of 15 years were the most affected, and boys were twice as likely as girls to be infected. The highest risk of disease was associated with the socio-economic circumstances of the individual student at home. The study indicated that mild to moderate tungiasis could be reduced by a third, and severe tungiasis by over half, if sleeping places of children had hardened floors, whilst approximately a seventh of the cases could be prevented by sealing classroom floors in schools, and another fifth by using soap for daily feet washing.

**Conclusion:**

There is a clear role for public health workers to expand the WASH policy to include washing of feet with soap in school-aged children to fight tungiasis and to raise awareness of the importance of sealed floors.

## Author summary

Tungiasis is a neglected tropical skin disease caused by penetrated sand fleas, the adult female of which burrows into the skin of the feet. The parasite rapidly expands its body size by a factor of 2000. The growth causes immense itching, inflammation, pain and debilitation. The current lack of good treatment methods means people attempt to remove the fleas themselves with non-sterile instruments causing more damage. Control efforts focus on prevention but there is little data to guide this in East Africa. The current study reinforces our previously published results on the household level from the same communities, indicating that prevention needs to focus on hardening the floors of resource-poor families and integrating daily foot washing with soap into water, hygiene and sanitation programs.

## Introduction

Sand flea disease (tungiasis) is a highly neglected parasitic skin disease which inflicts pain and suffering on millions of impoverished people in South America, the Caribbean and sub-Saharan Africa. Research on tungiasis is scant and most of the publications originate from South America [1] [2],very little work has been done in sub-Saharan Africa. This is partly because even though the disease bears all the hallmarks of a Neglected Tropical Disease [1] it was until very recently [3] not included in the World Health Organisation’s list of Neglected Tropical Diseases. This made it difficult for funding organisations to invest research funds into determining the disease ecology and consequently the development of urgently needed treatment and prevention tools. Most research currently undertaken on tungiasis is small-scale, supported by national and international non-governmental organizations and well-wishers, reinforced by community-based self-help groups and is hence highly resource-constrained [2].

The development of the juvenile stages of the sand flea *Tunga penetrans*, is similar to that of other Siphonaptera; they live off the host but depend during their development on loose, sandy soil [4, 5]. In contrast to other flea species, the adult female sand flea becomes permanently parasitic on its host, where it burrows into the skin and undergoes a dramatic growth, increasing its volume about 2000-fold within eight days [6]. Tungiasis affects mostly the feet and is associated with a pattern of debilitating morbidity [6]. Itching, pain, swelling, deep fissures, ulcers and abscess formation are symptoms of an acute inflammatory response to embedded fleas and bacterial superinfections of the lesions. Chronic infections result in chronic pain, disability, disfigurement and mutilation of the feet [6, 7, 8]. Children with tungiasis are often ridiculed by their peers and it has been shown that physical incapacity, mental strain and distress reduce quality of life [9].

Awareness of the public health importance of tungiasis has been growing in East Africa in recent years [10], but data on epidemiological characteristics, necessary for the planning and implementation of a control program, do not exist. The work presented here was part of a larger observational study performed in disease endemic areas in coastal Kenya and included simultaneously implemented cross-sectional household-based risk factor surveys recently published [11] and school-based surveys presented here. To the best of our knowledge, combined, the two studies provide the most comprehensive risk factor study to date on tungiasis for Sub-Saharan Africa.

## Methods

The aim of the school-based study was to complement the household-based study to investigate prevalence and risk factors for tungiasis and whether targeting school-going children for treatment and prevention might be a viable option. The specific objectives included: 1) determine the prevalence of tungiasis in selected rural schools in Malindi sub-county, 2) whether there are specific school factors associated with disease, 3) whether school or home present the greater risk and 4) if similar or different risk factors associated with the disease might be identified through a school-based survey.

### Study area and population

The study was performed in Malindi Sub-county, Kilifi County, eastern Kenya, where tungiasis is endemic and previous reports indicated disease prevalence in villages to range from 8% to 65% [11]. Malindi Sub-county is divided into two ecological zones: Kakuyuni Sub-location, a densely populated area in the coastal strip with tropical climate, and Malanga Sub-location, a less densely populated area inland with a much drier climate. Most rural homesteads in these areas consist of several mud-walled houses with a palm thatch roof and sandy floor. Domestic animals such as goats, cats, dogs and chickens walk freely on the compounds. Subsistence farming is the only activity practiced by most of the population to sustain their livelihood.

The cross-sectional study was implemented between August 5 and October 3, 2014 in five primary schools; two schools in Kakuyuni Sub-location (labelled as KS1 and KS2), and three schools in Malanga Sub-location (labelled as MS1, MS2, MS3). Schools were a minimum of 2 km apart with distinct catchment areas from where children originated. All except MS3 were public schools. The schools were purposely selected based on recommendation from the county Public Health Department as schools that were affected by tungiasis and no interventions had previously taken place in the catchment areas of these schools. The schools were located in the same communities as the household study. For the risk factor study a case: control design was used with a 1:1 ratio. The sample size calculation to yield results with 95% confidence limits, 80% power, an assumed prevalence of exposure of 40% among controls and least extreme odds ratio to be detected of 1.5, indicated the need for a sample of 776 students (388 cases, 388 controls). To allow for incomplete data sets and be able to adjust for confounders we increased this by 20%, aiming to interview 930 students (465 cases, 465 controls).

**Fig 1:**
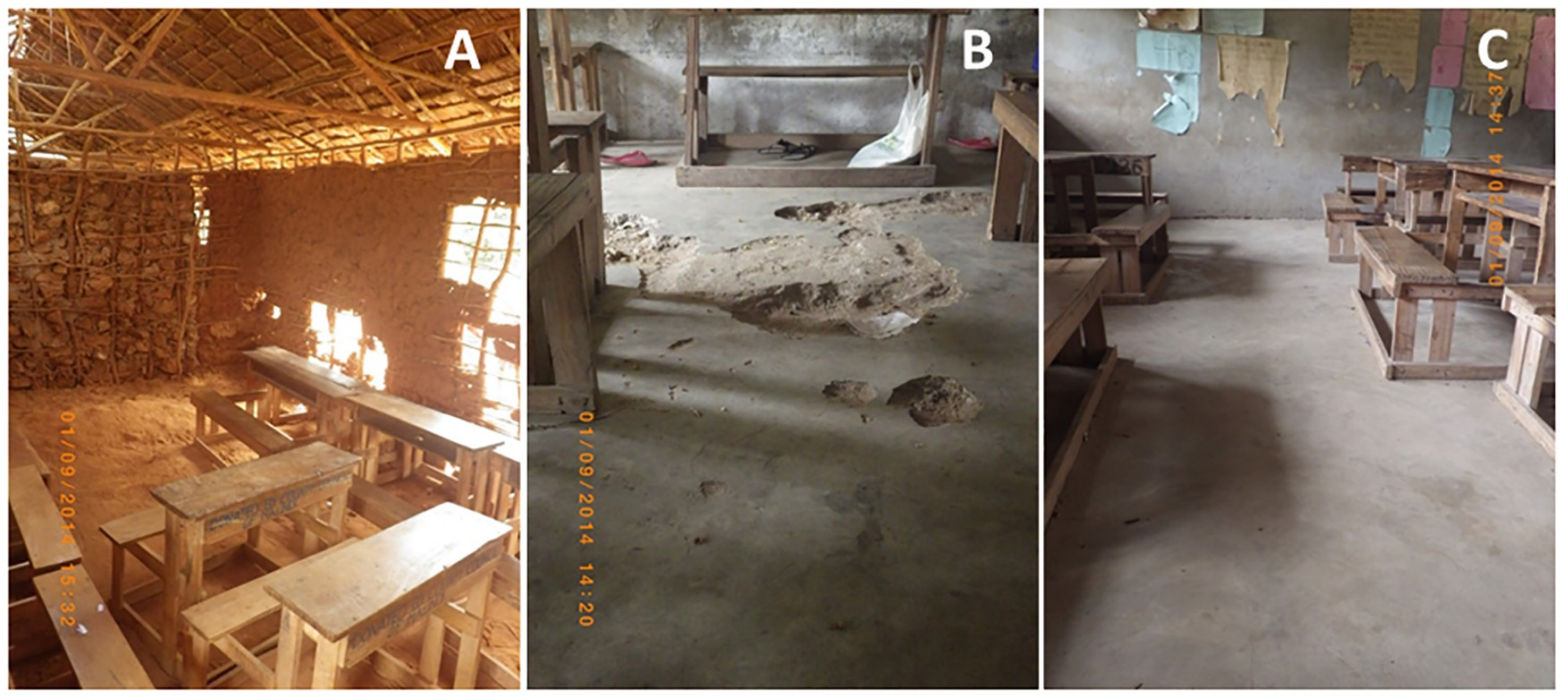
Examples of school classroom floors. (A) Natural sand/soil (MS2). (B) Cracked concrete (MS1). (C) Smooth concrete (MS1).

All schools were single-storey buildings and all were divided into several classrooms. MS2 had some classrooms of concrete and some of mud while all in MS3 were mud walls. The floor quality of the classrooms ranged from smooth cement surfaces to loose sand/soil (Fig 1). All schools had access to water and according to the information from the class teachers, classroom floors were swept daily by the students. For all classes, the classroom size, the number of students per class, and the type of classroom floor, walls and roof were recorded.

### Clinical examination

All female and male students of all age groups, for whom informed consent and assent was given, were clinically examined for tungiasis, class by class. Prior to the clinical examination, the feet of the students were carefully washed with soap in a basin. Each individual was then examined for tungiasis based on a standardized procedure [11], focussing on their feet and hands since a high number of lesions at the feet frequently coincide with the presence of ectopic lesions at the hands [12]. Patients were also asked whether they had tungiasis lesions in other regions of the body. Lesions were counted and staged according to the Fortaleza classification [6] as stage I: penetrating sand flea; stage II: brownish/black dot with a diameter of 1–2 mm surrounded or not by an erythema; stage III: circular yellow-white watch glass-like patch with a diameter of 3–10 mm and with a central black dot; stage IV: brownish-black crust with or without surrounding necrosis. Stage I to III are viable sand fleas. In stage IV the parasite is dying or already dead (non-viable). Lesions manipulated with a sharp instrument (by the patient or their caregiver) with the intention to remove the embedded parasite were documented as manipulated lesions. Based on the number of lesions present, the intensity of tungiasis was classified as light (1–5 lesions), moderate (6–30 lesions) or severe (>30 lesions) [13]. For every patient, the age, sex, and class were recorded.

To investigate potential changes in the presentation of tungiasis in students after school holidays we aimed to repeat the clinical examinations in as many students as possible in KS1 who had been originally examined in the week immediately before the 4-week August holiday. Only 248 of the original group of students examined could be traced in the first week after the holiday.

### Structured interviews

Within each school a subset of students was selected for interviews. In KS1 and MS1, all tungiasis cases over the age of 4 years and the following uninfected student (as an age-matched control) were interviewed. In the remaining schools, only the first half of the cases (in chronological order of their identification) and the following uninfected student, were interviewed due to time constraints. It was not possible to recruit equal numbers of controls for interviews in MS2 and MS3 because the majority of students had tungiasis. Structured interviews were conducted using a pre-tested questionnaire in Giriama or Swahili language, including questions about the physical structure of the house in which children slept (house walls, roof and floor), water sources and access at home, hygiene habits (washing frequency, soap use, toilet facility at home), livestock and companion animals kept in homestead and walking time to school. Furthermore, observations were recorded about the condition of the students’ school uniforms and the type of shoes worn if any.

### Statistical analysis

Generalized Estimating Equations were used to analyse potential associations between the prevalence of tungiasis or the number of lesions (of different stages) and multiple variables recorded during interviews and observations. Prevalence data were modelled using binomial probability distributions with logit link functions fitted, count data were modelled using negative binomial probability distributions with log link functions fitted. Depending on the analysis the unique school ID, the unique class ID or the unique student ID were included as repeated measure and an exchangeable correlation matrix assumed. In the final multivariable risk factor analyses only factors found significant when tested individually in a univariate analysis were included as predictors. Interactions were explored for variable combinations that were plausible to be potentially interacting. In the final model, only significant interactions were included. All reported mean proportions or mean counts and their 95% confidence intervals (CIs) were estimated as the exponentials of the parameter estimates for models with no intercept included. Frequency counts were compared using the Pearson Chi-Square test. The analyses were done with R statistical software version 2.14.2 [14]. Population Attributable Fractions (PAF) were calculated, representing the fraction of cases which would not have occurred if an exposure had been avoided, assuming the exposure is casual and the other risk factors in the population remain unchanged [15]. PAFs are the percent exposed among cases multiplied by the attributable risk (AR). The AR is the risk of tungiasis in the exposed due to the exposure and is calculated as (odds ratio (OR) −1)/OR.

### Ethical considerations

The study was approved by the Ethics Review Committee at Pwani University, Kilifi County, Kenya; approval number ERC/PhD/010/2014. During the study preparation phase contact was made with the County and District leadership in the Ministry of Health and the Ministry of Education, the Zonal Education Officer and the Community Health Officers to obtain their approvals and support for the study. Meetings were held with Community Health Workers (CHWs) in each sub-location, training on tungiasis provided and the aims and procedures of the study explained, emphasizing that participation was completely voluntary, and subjects had the opportunity to withdraw from the study at any point in the study. Head Teachers were visited and provided with an information sheet. The information was read out at a parents’ meeting prior to the study, explaining the procedure and voluntary nature of participation, and asking for consent. The Head Teacher signed the consent form (S1 Annex) on behalf of the parents and school. Data were collected with the help of Community Health Volunteers of the respective Community Health Unit. All data analyses were conducted anonymously.

All students with tungiasis were treated after the survey by the Community Health Workers according to standard practice in Kilifi County [10]. For those with secondary bacterial infection and other illnesses requiring treatment, a referral form was prepared by a Community Health Worker, and patients were referred to the nearest Health Facility.

## Results

**Fig 2:**
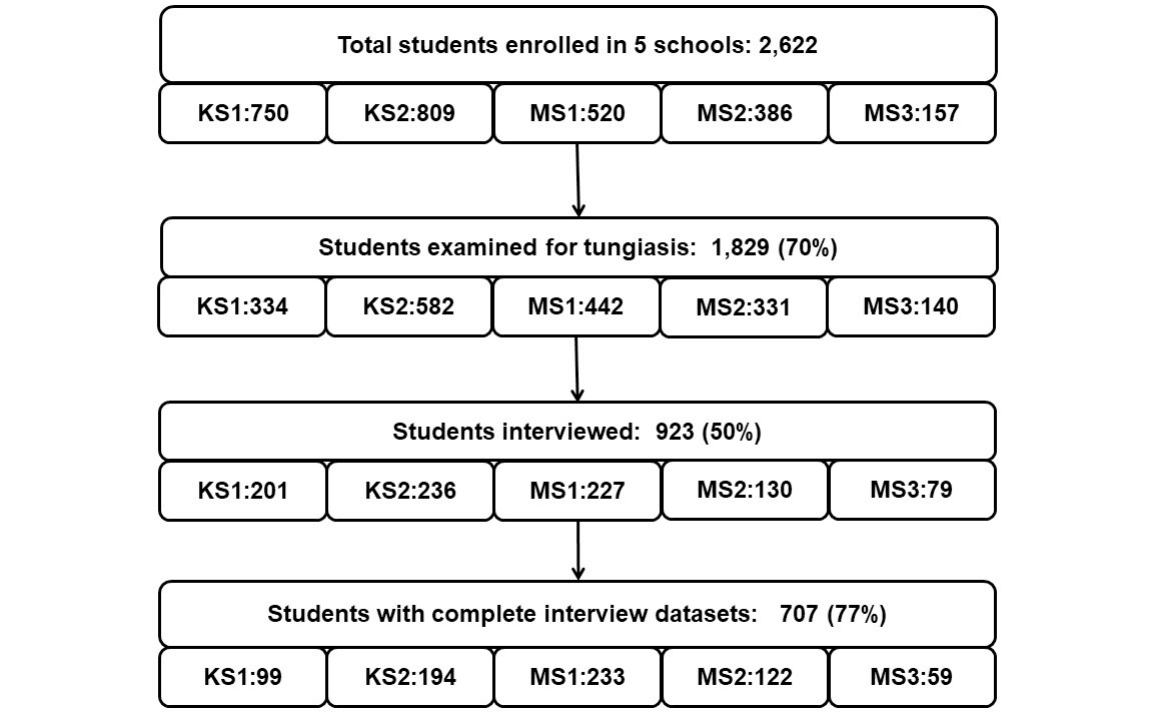
Flow chart of study population.

A total of 1,829 students from 56 classes in the 5 schools were examined, about 70% of the students enrolled in the schools (total 2,622) based on school records. Of these, 923 students were interviewed, just short of the calculated sample size of 930, but only 707 had fully completed the interview and were included in the data analysis; 398 were cases and 309 were controls (Fig 2).

### Prevalence of tungiasis and intensity of infection

**Fig 3:**
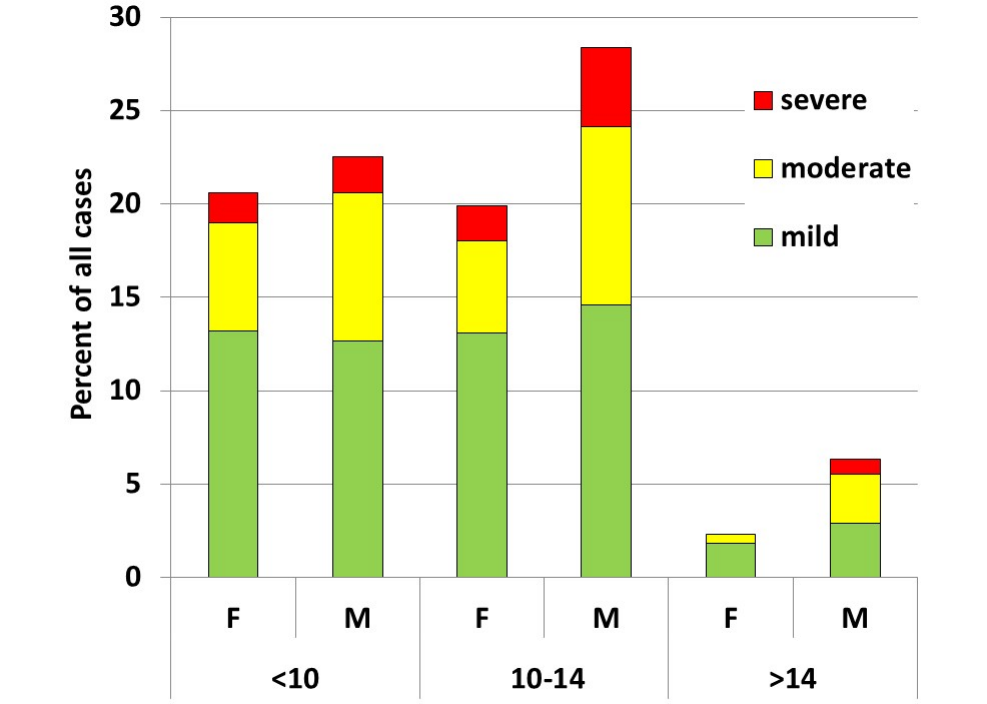
Severity of tungiasis by age group and sex. n = 870 cases; viable, non-viable and manipulated lesions combined. Mild < 6 fleas, moderate = 6–30 fleas and severe >30 fleas.

Of the examined 1,829 primary school students 48% were boys and 52% were girls; 31% of the students were 2–9 years old, 52% 10–14 years old and 17% were 15–21 years old. Of the 870 (48% of 1,829) students with tungiasis, 58% had mild infections (1–5 lesions), 31% moderate (6–30 lesions) and 11% severe infections (>30 lesions;). The majority of all cases were males aged 10–14 years (28.4% of cases, Fig 3) and also had the highest percent of moderate and severe cases. The student population size examined ranged from 140 to 582 between schools and the prevalence of tungiasis varied significantly between the five schools ranging from 31% to 83% (Table 1).

**Table 1:** Multivariable analysis for tungiasis prevalence in the five schools surveyed.

Parameter	Total examined (n)	Modelled mean tungiasis prevalence (%)	95% Confidence Interval (CI)		Odds Ratio (OR)	95% Confidence Interval (CI)		*p*
			Lower	Upper		Lower	Upper	
**School**								
MS3	140	83	75	88	2.54	0.85	7.57	0.094
MS2	331	75	68	82	1.88	0.82	4.32	0.139
MS1	442	34	29	38	0.77	0.48	1.24	0.279
KS2	582	51	37	65	2.35	0.82	6.76	0.112
KS1	334	31	26	36	1.00			
**Classroom floor**								
natural sand/soil	268	80	74	85	3.00	1.15	7.79	0.024
cracked concrete	725	49	43	55	0.51	0.30	0.87	0.014
smooth concrete	836	46	38	55	1.00			
Interaction school * classroom floor								
MS3*natural sand/soil	140	83	75	88	1.00			
MS2*natural sand/soil	128	78	67	86	1.00			
MS2*cracked concrete	69	87	77	94	11.54	3.30	40.37	<0.001
MS2*smooth concrete	134	54	36	72	1.00			
MS1*cracked concrete	96	35	30	40	2.16	1.13	4.13	0.020
MS1*smooth concrete	346	33	26	40	1.00			
KS2*cracked concrete	370	43	31	56	0.99	0.30	3.32	0.987
KS2*smooth concrete	212	60	35	81	1.00			
KS1*cracked concrete	190	24	18	32	1.00			
KS1*smooth concrete	144	39	31	47	1.00			
**Sex**								
Male	874	65	60	71	2.37	1.89	2.97	<0.001
Female	955	44	40	49	1.00			
**Age**								
2–9 years	571	60	54	66	1.57	1.04	2.36	0.032
10–14 years	942	56	50	62	1.35	1.07	1.69	0.011
15–21 years	316	49	41	57	1.00			

The physical school environment was considered a potential risk factor for tungiasis either directly providing a conducive environment for the off-host host stages to develop and adult sand fleas to find a host or indirectly as a proxy measure for the socio-economic circumstances affecting the community from which the children were drawn to the respective schools (children from poor background might only be able to afford sending children to a school with very basic school environment). Size, materials of the floors, walls and roofs were recorded for every classroom from which children were examined during the surveys. The majority (70%) of the students were taught in classrooms between 40–70 m^2^ while 22% were taught in classrooms larger than 70 m^2^ and only 8% in classrooms smaller than 40 m^2^. Classroom size was not significantly associated with tungiasis prevalence in a univariate analysis, and neither was the number of children per m^2^ in a classroom, which ranged from 0.4 to 2.0.

Most classroom floors were made of concrete; 46% of all screened students were taught in a room with a good quality concrete floor and 40% in a classroom with cracked concrete floor. Concrete floors were always associated with concrete walls and iron sheet roofs. Only 14% of the surveyed students studied in classrooms with a natural sand/soil floor. These classrooms also had mud walls and thatched roofs (Fig 1).

The differences in tungiasis prevalence between schools were confounded by the physical classroom environment as revealed by the multivariable analysis (Table 1). The floor type of the classroom was an important risk factor for finding a tungiasis case, the degree of risk per floor type, however, was dependent on the school, as shown by the significant interaction between floor type and school. Natural sand or soil floor of a classroom was an independent risk factor, increasing the probability of finding a tungiasis case 3-fold as compared to finding a case among students that were taught in a classroom with a well-kept, smooth concrete floor. Classrooms with natural sand/soil floors were only present in MS2 and MS3, the two schools with the highest tungiasis prevalence. Whether a cracked concrete floor represented a risk for finding a tungiasis case depended significantly on the school. The impact of the interactions can be calculated by multiplication of the odds ratios [16]. This means a cracked concrete floor was only a factor significantly associated with increased disease risk in MS2 (OR 11.54 x OR 0.51 = OR 5.89), where classrooms with sand/soil and concrete floors coexisted but not in MS1 (OR 2.16 x OR 0.51 = OR 1.10; Table 1). Consequently, floor type was not an explanatory variable for tungiasis prevalence in KS1, KS2 and MS1, where tungiasis prevalence ranged between 31% and 51% (Table 1).

Both age and sex were significantly, and independently associated with tungiasis. Children below the age of 15 years were 1.4–1.6 times more likely to be diagnosed with tungiasis than older students and boys more than 2 times more likely than girls (Table 1).

No specific school-based risk factors were significantly associated with severe tungiasis, when the multivariable analysis was repeated with severe manifestation as the dependent variable.

### Comparison of tungiasis prevalence and numbers of viable, non-viable and manipulated lesions before and after school holidays

Anecdotal information provided by the teachers suggested that children usually return to school with a higher tungiasis burden after school holidays. To investigate this, we compared the tungiasis prevalence and infection status immediately before and after the one-month August school holiday in the 248 students who were able to be traced in the first week after the holiday in KS1 (Tables 2 and 3). This sub-group comprised 41% boys, 59% girls, with 23% <10 years old, 55% 10–14 years old and 22% 15–20 years old.

**Table 2:** Multivariable analysis to investigate the association between school holidays and tungiasis prevalence in KS1.

Parameter	Total examined (n)	Modelled mean tungiasis prevalence (%)	95% Confidence Interval (CI)		Odds Ratio (OR)	95% Confidence Interval (CI)		*p*
			Lower	Upper		Lower	Upper	
**Sex**								
Male	202	50	40	60	3.07	1.89	5.01	<0.001
Female	294	26	19	33	1.00			
**Age**								
2–9 years	116	48	37	59	2.94	1.37	6.29	0.006
10–14 years	272	38	30	45	1.70	0.86	3.35	0.126
15–21 years	108	27	16	41	1.00			
**Survey**								
after holidays	248	44	36	51	1.71	1.31	2.23	<0.001
before holidays	248	31	25	38	1.00			

**Table 3:** Multivariable analyses investigating the risk of finding viable, non-viable and manipulated lesions after school holidays in KS1 as compared to the baseline survey.

Parameter	Total tungiasis cases analysed (n)	Modelled mean number of lesions*	95% Confidence Interval (CI)		Rate Ratio (RR)	95% Confidence Interval (CI)		*p*
			Lower	Upper		Lower	Upper	
**(A) Viable lesions**								
**Sex**								
Male	105	1.53	0.93	2.51	0.77	0.34	1.74	0.531
Female	79	1.32	0.79	2.23	1.00			
**Age**								
2–9 years	54	0.88	0.44	1.74	0.68	0.21	2.25	0.528
10–14 years	104	1.79	1.13	3.83	1.13	0.40	3.21	0.819
15–21 years	26	1.83	0.86	3.89	1.00			
**Survey**								
after holidays	106	0.80	0.50	1.29	0.30	0.10	0.84	0.023
before holidays	78	2.52	1.61	3.93	1.00			
Sex*Survey								
male*after holidays	58	1.06	0.60	1.86	2.24	0.86	5.84	0.098
male*before holidays	47	2.21	1.21	4.03	1.00			
female years*after holidays	48	0.61	0.30	1.23	1.00			
female*before holidays	31	2.87	1.56	5.27	1.00			
Age*Survey								
2–9 years*after holidays	32	0.41	0.15	1.10	0.49	0.12	2.05	0.331
2–9 years*before holidays	22	1.87	0.81	4.33	1.00			
10–14 years*after holidays	59	1.03	0.59	1.79	0.75	0.31	1.82	0.519
10–14 years*before holidays	45	3.10	1.79	5.37	1.00			
15–21 years *after holidays	15	1.22	0.54	2.76	1.00			
15–21 years*before holidays	11	2.75	1.15	6.50	1.00			
**(B) Non-viable lesions**								
**Sex**								
Male	105	4.03	2.87	5.68	1.18	0.72	1.94	0.519
Female	79	3.42	2.29	5.12	1.00			
**Age**								
2–9 years	54	3.30	1.94	5.61	2.62	0.83	8.29	0.101
10–14 years	104	5.33	3.97	7.17	4.46	1.59	12.46	0.004
15–21 years	26	2.92	1.53	5.23	1.00			
**Survey**								
after holidays	106	3.06	2.19	4.27	2.14	0.63	7.34	0.225
before holidays	78	4.52	3.02	6.75	1.00			
Age*Survey								
2–9 years*after holidays	32	2.09	1.19	3.67	0.19	0.05	0.72	0.015
2–9 years*before holidays	22	5.22	2.79	9.74	1.00			
10–14 years*after holidays	59	3.21	2.17	4.74	0.17	0.04	0.66	0.01
10–14 years*before holidays	45	8.87	5.76	13.68	1.00			
15–21 years *after holidays	15	4.27	2.06	8.85	1.00			
15–21 years*before holidays	11	1.99	0.77	5.16	1.00			
**(C) Manipulated lesions**								
**Sex**								
Male	105	9.18	6.87	12.26	2.61	1.44	4.74	0.002
Female	79	5.40	4.04	7.21	1.00			
**Age**								
2–9 years	54	6.52	4.80	8.85	0.90	0.38	2.11	0.807
10–14 years	104	7.30	5.54	9.62	0.67	0.29	1.56	0.357
15–21 years	26	7.33	4.41	12.17	1.00			
**Survey**								
after holidays	106	10.02	8.23	12.20	2.41	1.19	4.90	0.015
before holidays	78	4.94	3.57	6.84	1.00			
Sex*Survey								
male*after holidays	58	10.55	7.86	14.16	0.42	0.21	0.85	0.016
male*before holidays	47	7.98	5.35	11.91	1.00			
female years*after holidays	48	9.52	7.24	12.53	1.00			
female*before holidays	31	3.06	1.89	4.94	1.00			
Age*Survey								
2–9 years*after holidays	32	8.09	5.56	11.78	0.98	0.44	2.17	0.961
2–9 years*before holidays	22	5.25	3.36	8.21	1.00			
10–14 years*after holidays	59	13.55	10.15	18.08	2.19	1.08	4.45	0.03
10–14 years*before holidays	45	3.94	2.55	6.08	1.00			
15–21 years *after holidays	15	9.19	6.36	13.27	1.00			
15–21 years*before holidays	11	5.84	2.81	12.13	1.00			

*modelled for cases (students with any lesions) only

Sex and age were identified, similar to the analysis on the larger data set, as independent risk factors for tungiasis before and after the school holidays, with boys and younger age groups more likely to be found with the disease than girls and older age groups (Table 2). There was no significant interaction between sex, age and survey time. The probability of finding a tungiasis case after the school holidays was 1.7 times higher than before the holidays, with a mean prevalence in students of 31% (95% CI 25–38%) before and 44% (95% CI 36–51) after the holidays (Table 2).

Taking a closer look at the number and developmental stage of the embedded sand fleas (Table 3), we observed that the number of viable lesions in students with tungiasis had significantly decreased (RR 0.30 (95% CI 0.10–0.84), p = 0.023) after the holidays from a mean number of 2.52 (95% CI 1.61–3.93) viable lesions before to 0.80 (95% CI 0.50–1.29) after the holidays, irrespective of sex and age. The number of non-viable lesions was generally highest in students with tungiasis between 10–14 year of age irrespective of sex and survey round, however significantly decreased (RR 0.17 (95% CI 0.04–0.66), p = 0.010) after the holidays from a mean of 8.87 to a mean of 3.21 (Table 3). A proportionally similar decrease in non-viable lesions was also seen in the younger age group of 2–9 years old students but not in the older students as shown by the significant interactions between age and survey round (Table 3). On the contrary, the number of manipulated lesions increased significantly after the school holidays. Significant interactions in the analysis (Table 3) highlighted a proportionally higher increase in manipulated lesions in girls from 3.06 (95% CI 1.84–4.94) before to 9.52 (95% CI 7.24–12.53) after holidays, than in boys, even though boys had overall a larger number of manipulated lesions (mean before holidays 7.98 (95% CI 5.35–11.91); after holidays 10.55 (95% CI 7.86–14.16)). There was also a proportionally higher increase in the number of manipulated lesions in the 10-14-year-old students than in the other age groups (Table 3).

### Behavioural and socio-economic tungiasis risk factors identified from structured student interviews and observations

Most of the characteristics assessed by observation and interview, for each of the 707 students interviewed, showed considerable heterogeneity between schools, except for sex and ownership of dogs and chickens (Table 4).

**Table 4:** Frequency of individual and household characteristics identified by interview and observation per school (*p = Pearson Chi-Square).

Explanatory variables	Variable categories	Percent (%) of all students interviewed per school					Total n = 707	p*
		KS1	KS2	MS1	MS2	MS3		
		n = 99	n = 194	n = 233	n = 122	n = 59		
**Sex**	female	44	44	49	38	54	46	0.212
	male	56	56	51	62	46	54	
**Age group**	2–9 years	3	26	16	30	68	24	<0.001
	10–14 years	70	60	69	51	32	60	
	15–21 years	27	15	15	19	0	16	
**Shoes worn to school**	closed shoes	14	7	10	6	3	9	<0.001
	open shoes	61	56	54	31	36	50	
	no shoes	25	37	36	63	61	41	
**Walking time to school**	less 30 minutes	54	54	63	63	51	58	<0.001
	more 30 minutes	35	34	37	36	29	35	
	no answer	11	12	0	1	20	7	
**Condition of uniform**	good	7	14	58	69	70	42	<0.001
	moderate	70	73	40	27	31	50	
	torn	23	13	1	4	0	8	
**Material of home floor**	stone/cement	25	15	10	5	12	13	<0.001
	smeared mud	19	23	37	13	17	25	
	sand	56	62	53	83	71	62	
**Material of home walls**	stone/cement	25	17	5	4	0	11	<0.001
	smeared mud	74	83	94	95	100	89	
	palm leaves	1	1	1	1	0	1	
**Material of home roof**	thatched	61	51	54	64	44	55	0.042
	corrugated iron sheets	39	49	46	36	56	45	
**Source of water at home**	tap on compound	34	39	7	8	5	20	<0.001
	shared community tap/ well	57	62	93	92	93	79	
	other	9	0	0	0	2	1	
**Time to fetch water at home**	less than 15 minutes	80	82	72	58	58	72	<0.001
	more than 15 minutes	12	8	28	41	19	22	
	no answer	8	9	0	2	24	6	
**Frequency of washing feet per day**	twice a day	70	70	83	74	70	75	<0.001
	once a day	18	25	16	13	5	17	
	less often	6	5	2	0	0	3	
	no answer	6	1	0	13	25	5	
**Frequency of using soap for washing feet**	always	66	56	25	16	24	38	<0.001
	sometimes	24	39	72	68	49	53	
	never	9	4	3	3	2	4	
	no answer	1	2	0	14	25	5	
**Type of toilet at home**	WC	14	16	8	6	10	11	<0.001
	latrine	36	56	57	53	54	53	
	bush	50	27	36	29	31	34	
	no answer	0	1	0	12	5	3	
**Household owns dog**	yes	34	24	32	30	27	30	0.352
**Household owns chicken**	yes	94	97	96	94	95	95	0.745

The significant between-group variations for schools was taken into consideration by including the school as a random factor in the subsequent multivariable analyses of the interview data (Table 5). Since tungiasis cases and healthy controls were matched by age at the enrolment stage, age was not a factor significantly associated with tungiasis as an outcome in the analysis of the interview data and hence not included in the multivariable analysis. Expectedly, sex was similarly associated with tungiasis risk in the interview data, with boys being >2 times more likely to be found with tungiasis than girls. The condition of the school uniform was not independently associated with tungiasis, but an interaction existed with sex. The chance of finding tungiasis was significantly higher (OR 4.30 (95% CI 1.47–12.60), p = 0.008) in boys with torn school uniforms than in boys with better uniforms or in girls with torn uniforms (Table 5).

**Table 5:** Multivariable analysis exploring the association of observational and interview data with tungiasis outcome.

Explanatory variables	Total interviewed (n)	Modelled mean tungiasis prevalence (%)	95% Confidence Interval (CI)		Odds Ratio (OR)	95% Confidence Interval (CI)		*P*
			Lower	Upper		Lower	Upper	
**Sex**								
male	381	79	66	88	2.55	1.25	5.20	0.010
female	326	47	31	64	1.00			
**Condition of uniform**								
torn	55	67	44	84	0.43	0.12	1.55	0.197
moderate	356	68	51	81	1.54	0.83	2.85	0.172
good	296	59	47	71	1.00			
Sex*condition of uniform								
male*torn	36	86	72	95	4.30	1.47	12.60	0.008
male*moderate	215	77	61	88	0.99	0.44	2.25	0.985
male*good	130	70	56	81	1.00			
female*torn	19	38	18	63	1.00			
female*moderate	141	57	41	72	1.00			
female*good	166	48	33	63	1.00			
**Shoes worn during survey (observed)**								
none	293	78	60	89	1.61	0.67	3.87	0.289
open	353	61	46	74	1.80	0.70	4.63	0.223
closed	61	53	35	71	1.00			
Interaction clothes condition*shoes worn								
torn*none	25	89	70	96	5.32	3.22	8.79	<0.001
torn*open	26	53	38	68	0.69	0.15	3.10	0.629
torn*closed	4	48	19	78	1.00			
moderate*none	152	77	57	89	1.31	0.78	2.20	0.301
moderate*open	169	64	49	78	0.64	0.29	1.41	0.270
moderate*closed	35	61	41	78	1.00			
good*none	116	62	52	72	1.00			
good*open	158	65	49	78	1.00			
good*closed	22	51	30	71	1.00			
**Walking time to school**								
no answer	47	66	46	82	1.08	0.79	1.49	0.620
>30 minutes	247	64	50	76	0.98	0.69	1.39	0.918
<30 minutes	413	64	49	77	1.00			
**Material of floor at home**								
sand	439	70	56	82	1.88	1.64	2.15	<0.001
smeared mud	178	68	50	81	1.65	1.26	2.15	<0.001
stone/concrete	90	56	40	71	1.00			
**Material of wall at home**								
matts from plam leaves	5	59	31	82	0.61	0.22	1.69	0.342
mud	628	64	53	74	0.75	0.50	1.11	0.147
stone/concrete	74	71	55	83	1.00			
**Material of roof at home**								
thached	389	67	50	80	1.17	0.85	1.60	0.332
corrugated iron sheet	318	63	47	77	1.00			
**Source of water at home**								
other	10	52	25	77	0.56	0.25	1.27	0.165
community tap/well	559	76	65	84	1.63	1.44	1.85	<0.001
tap on compound	138	65	55	74	1.00			
**Time to fetch water at home**								
no answer	43	68	57	76	1.21	0.64	2.30	0.564
>15 minutes	153	63	43	80	0.99	0.64	1.52	0.959
<15 minutes	511	63	42	81	1.00			
**Number of times feet washed**								
no answer	40	87	80	91	6.01	3.64	9.92	<0.001
less than daily	19	55	36	72	1.12	0.62	2.02	0.705
once a day	121	57	37	76	1.24	0.68	2.28	0.480
twice a day	527	52	32	72	1.00			
**Frequency of soap use**								
no answer	38	37	25	50	0.47	0.25	0.89	0.020
never	28	89	70	96	6.33	3.17	12.64	<0.001
sometimes	377	67	52	79	1.62	1.50	1.76	<0.001
always	264	55	41	69	1.00			
**Type of toilet at home**								
no answer	20	76	28	96	2.11	0.18	25.17	0.555
bush	237	60	48	71	1.02	0.57	1.82	0.957
latrine	374	61	48	73	1.05	0.71	1.56	0.812
water closet	76	60	41	77	1.00			
**Dog at home**								
present	209	70	52	83	1.63	0.93	2.85	0.085
absent	498	59	42	74	1.00			
**Student’s classroom floor**								
natural sand/soil	112	78	64	88	3.08	2.39	3.97	<0.001
cracked concrete	291	59	44	73	1.24	1.12	1.37	<0.001
smooth concrete	304	54	37	70	1.00			

Similarly, the absence or presence of open or closed shoes was not by itself a risk factor for tungiasis. When a child was however found not wearing shoes and had a badly torn school uniform it was highly likely (OR 5.32 (95% CI 3.22–8.79), p<0.001) to find tungiasis (Table 5). The time a child took to walk to school was not significantly associated with presence of tungiasis in the multivariable analysis. Whilst the building material of the students’ homes floors, walls, and roofs were all associated with tungiasis in a univariate analysis only the home’s floor was an independent risk factor in the multivariable analysis. A student from a home with a natural sand or mud floor indoors was nearly twice as likely to be diagnosed with tungiasis, than a student from a home with a concrete floor in the house (Table 5). Most of the students had access to piped water or a well either on their compound or shared in the village. Nevertheless, students coming from a home where water was fetched from a community tap or well were 1.6 times more likely to have tungiasis than those students that had tap water on their compound at home. The time it takes for the family to fetch water was not associated with the disease outcome. Whilst the frequency of washing feet was not associated with the presence of tungiasis, the use of soap strongly was. Students that responded never to wash their feet with soap were over 6 times more likely (95% CI = 3.2–12.6, p<0.001) to have tungiasis and students that responded to only sometimes wash they feet with soap were 1.6 times more likely (95% CI 1.50–1.76, p<0.001) to have tungiasis than those students always washing with soap (Table 5). Although washing frequency responses were not significantly associated with tungiasis, not answering this question was (OR 6.01 (95% CI = 3.64–9.92), p<0.001). Neither the type of toilet at home, nor the presence of a dog on the compound, were significantly associated with disease outcome when the analysis was adjusted for all other variables.

In an attempt to better understand what drives severe infection (>30 embedded lesions) we performed a multivariable analysis for severe disease as the outcome (n = 71, 18% of all cases N = 398), comparing the characteristics of these severe cases with mild to moderate cases (1–30 lesions, n = 327). Sex, condition of clothing, shoe-wearing and frequency of soap use were not significantly associated with disease severity. Of all tungiasis cases, severe infections were more likely to be found in the younger age groups (Table 6), in children having a natural sand/mud floor indoors at home than those that have a stone/cement floor at home, using a water source other than a private or community tap or well, and washing their feet less than once a day. Of all children with tungiasis, those that reported their family did not own at least one chicken had a significantly higher risk of heavy infection than children who reported they kept chicken (Table 6). Amongst all children with tungiasis, school floor characteristics were not a predictor for heavy infection.

**Table 6:** Multivariable analysis exploring the association of observational and interview data with severe tungiasis amongst all tungiasis cases.

Parameter	Total cases interviewed (n)	Modelled mean percentage (%) of severe tungiasis cases*	95% Confidence Interval (CI)		Odds Ratio (OR)	95% Confidence Interval (CI)		*p*
			Lower	Upper		Lower	Upper	
**Age group**								
5–9 years	105	29	13	53	1.40	1.01	1.93	0.042
10–14 years	233	35	19	55	1.80	1.05	3.08	0.034
15–21 years	60	23	10	45	1.00			
**Material of floor at home**								
sand	261	40	23	60	3.10	1.48	6.49	0.003
smeared mud	100	31	16	52	2.05	1.12	3.74	0.020
stone/cement	37	18	6	43	1.00			
**Source of water at home**								
other	5	56	31	78	6.03	3.32	10.95	<.0001
community tap/well	333	20	11	34	1.18	0.53	2.64	0.680
tap on compound	60	17	6	42	1.00			
**Number of times feed washed**								
no answer	33	7	1	44	0.20	0.03	1.44	0.110
less than daily	11	62	45	76	4.19	1.87	9.38	<.0001
once a day	72	35	22	50	1.37	0.82	2.28	0.234
twice a day	282	28	16	44	1.00			
**Chicken at home**								
present	379	19	9	36	0.34	0.20	0.58	<.0001
absent	19	41	19	67	1.00			

*out of all tungiasis cases interviewed

### Population attributable fractions (PAF)

For those factors which were significant risks for tungiasis or a high intensity of infection, and are amenable to being changed, the PAF were calculated. The PAF is the percent reduction in prevalence that would occur if exposure to the risk factor were removed. The highest PAF for both, any infection and severe infection was found to be having a home floor of sand or smeared mud (30.7% and 54.4% respectively, Table 7). Only using soap sometimes when washing had a PAF of 21.5%, and a classroom floor of sand had a PAF of 14.3% for any type of infection.

**Table 7:** Population Attributable Fractions. (OR: Odds Ratio, AR: Attributable Risk, PAF: Population Attributable Fraction).

Factor	Any Infection				Heavy Infection			
	OR	AR	% exposed among cases	PAF (%)	OR	AR	% exposed among cases	PAF (%)
Classroom floor of sand	2.99	0.67	21.5	14.3				
Home floor of sand	1.88	0.47	65.6	30.7	3.1	0.68	80.3	54.4
Home floor smeared mud					2.05	0.51	16.9	8.7
Never using soap for washing feet	6.3	0.84	6.1	5.1				
Only using soap for washing sometimes	1.6	0.38	57.2	21.5				
other water source					6.03	0.83	4.2	3.5
wash less than daily					4.19	0.76	5.6	4.3
Do not own a chicken					5.19	0.81	9.9	8.0

## Discussion

Our study confirmed that the prevalence of tungiasis is extremely heterogeneous, varying from school to school and community to community even though they are only a few kilometres apart [17]. The disease burden was highly aggregated even within an individual school, with more than half of the cases having only a few embedded sand fleas but a minority being severely affected. The overall prevalence of 48% of all screened school-aged children was twice as high as the prevalence in the simultaneously implemented household study [11], reflecting the high proportion of the most affected age group in the school-based study. As has been shown before in Brazil [17], Uganda [18], Nigeria [19], and Kenya [20, 21], school-aged children below the age of 15 years were the most affected by tungiasis and boys were twice as likely as girls to have the disease.

The risk factor interviews as well as the follow up examinations after the long school holidays suggest strongly that the highest risk of disease is associated with the socio-economic circumstances of the individual student at home. Whilst an unsealed, natural sand or soil floor of a classroom came out as an independent risk factor in the analysis it is important to note that such classroom floors were only present in two schools where the majority of pupils came from homes that had unsealed floors. The calculation of the PAF indicates that mild to moderate tungiasis could be reduced by a third, and severe tungiasis by over a half, if homes (sleeping places of children) had sealed floors, whilst approximately a seventh of the cases could be prevented by sealing classroom floors in schools. The presence of unsealed floors at home, has been previously indicated as an important risk factor for the disease [13, 21, 22], and can only be a consequence of the biology of the sand flea, with egg, larval and pupal (off-host stages) development taking approximately three weeks and requiring shaded, dry, loose soil or sand [4, 5]. Such unsealed floors provide a constant supply of the sand fleas searching for hosts as soon as they emerge as adults. This finding also corroborates the assumption that in settings where the prevalence of tungiasis is stable the whole year round, the transmission mainly takes place inside the house, particularly in the room where children sleep [1].

Neither the condition of the school uniform, nor wearing shoes potentially protecting against host-seeking sand fleas [23] was independently associated with tungiasis, however, the combination of a torn uniform and the absence of shoes can be considered an indicator of the poverty level or care given to the child at home. Complex interactions between risk factors suggest underlying behavioural differences in the care given by parents and guardians and/or hygiene behaviour expressed in boys and girls. Boys with torn uniforms were four times more likely to be affected by the disease than girls wearing uniforms in equally poor condition.

Whilst the frequency of washing was not associated with tungiasis, the availability of piped or well water within the homestead and the use of soap when washing was strongly associated with reduced risk. Both factors might be linked to the socio-economic status of the family to afford piped water and soap, but also to behavioural characteristics of the care givers and children, as already noted in the household study [18].

The higher risk of infection and severe disease observed for boys between the ages of 10 and 14 years may be a reflection of their hygiene practices, being less likely to wash daily, particularly with soap than girls in the same age group. Whilst such a sex-specific association was not detected in the analysis in the current study, previous studies using interviews and observation in other countries have found boys to have poorer skin hygiene than girls [24]. A recent study examining the relationship between socioeconomic status and WASH practices in India also highlighted the fact that over 80% of mothers did use soap to wash themselves but only 20% used soap to wash their children [25].

The data from before and after school holidays, whilst a small dataset, highlighted a number of findings that are significant and warrant replication in future. Not only did the overall prevalence of tungiasis increase after the holiday, there was also a significant increase in the number of manipulated lesions. These are the sores and cicatrices that remain after an embedded sand flea has been purposively extracted with a sharp instrument and are a clear indication that the person recently had a viable embedded sand flea. The likely explanation is that the children have acquired more sand fleas whilst spending more time at home during the holiday, but they or a caregiver have extracted them. There was a significant interaction between the number of manipulated lesions and girls, again suggesting differential hygiene and caring behaviours. Teasing apart these complex linkages of economic status and behavioural traits will be important in future studies and might indicate school-aged boys to be an important target for prevention programs.

Surveys in other countries have identified tungiasis as a zoonosis with the involvement of dogs [13] and pigs [26, 27] in disease transmission. Pigs are not frequently kept in communities in coastal Kenya (only four students in the survey reported owning pigs), whilst goats and dogs were relatively common with 75% and 30% of students reporting household ownership, respectively. However, neither the previously published household study [11] nor the here presented school survey identified the possession of any animal species to be a risk factor. Whether this is an indication that transmission in these coastal communities is purely intra-domiciliary and does not involve an animal reservoir needs further investigation by examining livestock and companion animals for tungiasis. The current school survey did identify the absence of chickens in a household as a risk factor for severe disease, which may simply be another reflection of extreme poverty as a risk factor, which needs to be studied however more systematically.

The fact that the same household risk factors were identified in this study by asking the children about their homes, as in the corresponding household study where adults were interviewed, and observations made, suggests that school-based surveys are a reasonable alternative to the more expensive and time-consuming household surveys and can be used for nation-wide evaluation of tungiasis prevalence. Modelling based on past household surveys with full age profiles will enable extrapolation to the whole population. School-based surveys have the advantage of a high concentration of at-risk subjects to survey during day time when there is good light for examinations. To be able to examine all house occupants for tungiasis a team must visit during evening hours and at weekends, and still many family members may be absent. Houses may be far apart, and therefore surveying costly to achieve suitable sample sizes.

Targeting school-aged children in school for diagnosis and treatment of tungiasis, using recently evaluated safe and effective treatment options, namely dimeticone or neem oil [2, 28, 29], might be the most cost-effective way to reduce the disease burden given that the affected resource-poor communities do not have access to optimal medical care and limited ability to pay for expensive medications. However, the treatment must be provided every time a new infection in a child is detected by the teachers to prevent the life cycle being introduced into classrooms with a cracked or natural sand/mud floor, and to break the cycle at home. The disadvantage of conducting surveys and treatment programs only in schools means the most severely affected children who cannot walk to attend school, the elderly and disabled, who also tend to have severe infections, will be missed.

This factor was a possible limitation of the study, possibly causing bias in the study findings. However, the study included a similar proportion of severe cases to that seen in the household survey (11% and 15% respectively), so any effect on outcomes is likely to be minimal. The higher proportion of severe cases in the household survey was more likely to be due to the inclusion of the elderly who tend to have more severe infections [11].

Another limitation of the study was the low number of schools with non-cemented floors that were able to be included in the study, and that the one school that was entirely dirt floors, was the only private school, with the majorty of children in the lower age groups. However, any potential confounding was adjusted for in the statistical modeling.

Observations from our study suggest that up to 70% of tungiasis cases may be prevented through simple prevention methods, namely washing feet at least once a day with soap and installing hard floors in homes and schools. Hence, foot washing needs to be incorporated into hygiene and sanitation education campaigns of the current global efforts to achieve Sustainable Development Goal 6; “by 2030, achieve access to adequate and equitable sanitation and hygiene for all”. Tungiasis has been implicated to impact children’s learning capacity [9], consequently, there is a clear role and need for schools (head teachers and class teachers), public health officials, community health workers and NGOs to educate in and enforce good hygiene practices, particularly the use of soap for daily washing of feet. Furthermore, acknowledging that sealed classroom floors can contribute to tungiasis reduction, governments and education officials need to make the cementing of all classroom floors a priority, along with adequate water supplies and provision of soap for washing.

The installation of hardened floors in family homes is not as simple as it sounds, and requires research and potentially government investment. Those resource-poor, marginalized families affected by tungiasis cannot, under most circumstances, afford the cost of a cement floor, which in Kenya costs a minimum of $200 for a typical rural house of 6 x 4 m. In discussions with community members, it was highlighted that in the past communities used traditional methods for hardening floors such as regular smearing with a mix of soil and cow dung and termite mound soil, but these methods have ceased, and floors are no longer hardened (Elson, personal communication). Clearly there is a need for research into understanding why house floors are not hardened with the simple, cheap methods currently available, as well as developing alternative, locally available and affordable floor technologies that the most resource-poor families can install themselves.

## Supporting information

S1 Annex Information leaflet and consent form. (DOCX)

S2 Annex STROBE checklist. (DOCX)

S3 Annex Database-tungiasis examination and classroom characteristics. (XLSX)

S4 Annex Database-tungiasis before-after school holiday in KS1. (XLSX)

S5 Annex Database- tungiasis risk factor interviews. (XLSX)

## References

[1] Feldmeier H, Heukelbach J, Ugbomoiko U, Sentongo E, Mbabazi P, von Samson-Himmelstjerna G, Krantz I. A neglected disease with many challenges for global public health. PLoS Negl Trop Dis. 2014; 8(10): (e3133). 10.1371/journal.pntd.0003133 25356978 PMC4214674

[2] Elson L, Wright K, Swift J, Feldmeier H. Control of tungiasis in absence of a roadmap: grassroots and global approaches. Trop Med Infect Dis. 2017; 2: 33 10.3390/tropicalmed2030033 30270889 PMC6082108

[3] World Health Organization. [Internet]. Geneva: Neglected Tropical Diseases. [cited 2018 August 31]. Available from: http://www.who.int/neglected_diseases/diseases/en/.

[4] Nagy N, Abari E, D’Haese J, Calheiros C, Heukelbach J, Mencke N, et al Investigations on the life cycle and morphology of *Tunga penetrans* in Brazil. Parasitol Res. 2007; 101 (Suppl 2): S233–S242. 10.1007/s00436-007-0683-8 17823833

[5] Linardi P, Calheiros CM, Campelo-Junior E, Duarte E, Heukelbach J, Feldmeier H. Occurrence of the off-host life stages of *Tunga penetrans* (Siphonaptera) in various environments in Brazil. Ann Trop Med Par. 2010; 104(4): 337–45. 10.1179/136485910X12743554759902 20659395

[6] Eisele M, Heukelbach J, Van Marck E, Mehlhorn H, Meckes O, Franck S, Feldmeier H. Investigations on the biology, epidemiology, pathology and control of *Tunga penetrans* in Brazil: I. Natural history of tungiasis in man. Parasitol Res. 2003; 90(2): 87–99. 10.1007/s00436-002-0817-y 12756541

[7] Feldmeier H, Eisele M, Van Marck E, Mehlhorn H, Ribeiro R, Heukelbach J. Investigations on the biology, epidemiology, pathology and control of *Tunga penetrans* in Brazil: IV. Clinical and histopathology. Parasitol Res. 2004; 94: 275–282. 10.1007/s00436-004-1197-2 15368123

[8] Feldmeier H, Eisele M, Saboia-Moura R, Heukelbach J. Severe tungiasis in underprivileged communities: case series from Brazil. Emerg Infect Dis. 2003; 9: 949–955. 10.3201/eid0908.030041 12967492 PMC3020603

[9] Wiese S, Elson L, Feldmeier H. Tungiasis-related life quality impairment in children living in rural Kenya. PLoS Negl Trop Dis. 2017; 12(1): e0005939 10.1371/journal.pntd.0005939 29309411 PMC5757912

[10] Ministry of Health. [Internet]. Nairobi: National policy guidelines on prevention and control of jigger infestations. Pub: Division of Environmental Health; 2014. http://guidelines.health.go.ke/#/category/12/95/meta.

[11] Wiese S, Elson L, Reichert F, Mambo B, Feldmeier H. Prevalence, intensity and risk factors of tungiasis in Kilifi County, Kenya: I.Results from a community-based study. PLoS Negl Trop Dis. 2017; 11(10): e0005925 10.1371/journal.pntd.0005925 28991909 PMC5648262

[12] Heukelbach J, Wilke T, Eisele M, Feldmeier H. Ectopic localization of tungiasis. Am J Trop Med Hyg. 2002; 67(2): 214–216. 12389950 10.4269/ajtmh.2002.67.214

[13] Muehlen M, Feldmeier H, Wilcke T, Winter B, Heukelbach J. Identifying risk factors for tungiasis and heavy infestation in a resource-poor community in Northeast Brazil. Trans R Soc Trop Med Hyg. 2006; 100: 371–380. 10.1016/j.trstmh.2005.06.033 16297946

[14] R Core Team. R: A language and environment for statistical computing. Foundation for statistical computing. [Internet]. 2017 [cited 2018 August 31]. Available from: http://www.R-project.org/.

[15] Kleinbaum D, Kupper L, Mogernstern H. Epidemiologic research: principles and quantitative methods. Belmont (California), USA: John Wiley & Sons; 1982. ISBN: 978-0-471-28985-2

[16] Katz MH. Multivariable analysis: a practical guide for clinicians and public health researchers. Cambridge University Press; New York, USA Third Edition 2011. ISBN 978-0-521-14107-9

[17] Muehlen M, Heukelbach J, Wilcke T, Winter B, Mehlhorn H, Feldmeier H. Investigations on the biology, epidemiology, pathology and control of Tunga penetrans in Brazil.II. Prevalence, parasite load and topographic distribution of lesions in the population of a traditional fishing village. Parasitol Res. 2003; 90: 449–455. 12768415 10.1007/s00436-003-0877-7

[18] Wafula S, Ssemugabo C, Namuhani N, Musoke D, Ssempebwa J, Halage A. Prevalence and risk factors associated with tungiasis in Mayuge district, Eastern Uganda. Pan Afr Med J. 2016; 24: 77 10.11604/pamj.2016.24.77.8916 27642416 PMC5012786

[19] Ugbomoiko US, Ofoezie IE, Heukelbach J. Tungiasis: high prevalence, parasite load, and morbidity in arural community in Lagos State, Nigeria. Int J Dermatol. 2007; 46: 475–481. 10.1111/j.1365-4632.2007.03245.x 17472674

[20] Kimani B, Nyagero J, Ikamari L. Knowledge, attitude and practices on jigger infestation among household members aged 18 to 60 years: case study of a rural location in Kenya. Pan Afr Med J. 2012; 13: 7 23467785 PMC3589250

[21] Mwangi J, Ozwara H, Gicheru M. Epidemiology of *Tunga penetrans* infestation in selected areas in Kiharu constituency, Murang’a County, Kenya. Trop Dis Travel Med Vaccines. 2015; 1: 13 10.1186/s40794-015-0015-4 28883944 PMC5530937

[22] Ugbomoiko US, Ariza L, Ofoezie IE, Heukelbach J. Risk factors for tungiasis in Nigeria: identification of targets for effective intervention. PLoS Negl Trop Dis. 2007; 1: e87 10.1371/journal.pntd.0000087 18160986 PMC2154384

[23] Tomczyk S, Deribe K, Brooker SJ, Clark H, Rafique K, Stefanie K, Utzinger J, Davey G. Association between footwear use and Neglected Tropical Diseases: a systematic review and meta analysis. PLoS Negl Trop Dis. 2014; 8(11): e3285 10.1371/journal.pntd.0003285 25393620 PMC4230915

[24] Mhaske MS, Khismatrao DS, Fernandez K, Pandve HT, Kundap RP. Morbidity pattern and personal hygiene in children among private primary school in urban area: are the trends changing? J Family Med Prim Care. 2013; 2(3): 266–269. 10.4103/2249-4863.120753 24479095 PMC3902684

[25] Raihan M, Farzana F, Sultana S, Haque M, Rahman A, Waid J. Examining the relationship between socio-economic status, WASH practices and wasting. PLoS ONE. 2017; 12(3): e0172134 10.1371/journal.pone.0172134 28278161 PMC5344309

[26] Cooper J. An outbreak of *Tunga penetrans* in a pig herd. Vet Rec. 1967; 80: 365–366. 4166051 10.1136/vr.80.11.365

[27] Mutebi F, Krücken J, Feldmeier H, Waiswa C, Mencke N, Sentongo E, von Samson-Himmelstjerna G. Animal reservoirs of zoonotic tungiasis in endemic rural villages of Uganda. PLoS Negl Trop Dis. 2015; 9(10): e0004126 10.1371/journal.pntd.0004126 26473360 PMC4608570

[28] Nordin P, Thielecke M, Ngomi N, Mudanga G, Krantz I, Feldmeier H. Treatment of tungiasis with a two component dimeticone: a comparisonbetween moistening the whole foot and directly targeting the embedded sand fleas. Trop Med Health. 2017; 45: 6 28293130 10.1186/s41182-017-0046-9 PMC5345134

[29] Thielecke M, Nordin P, Ngomi N, Feldmeier H. Treatment of tungiasis with dimeticone: a proof-of-principle study in rural Kenya. PLoS Negl Trop Dis. 2014; 8(7): e3058 10.1371/journal.pntd.0003058 25079375 PMC4117482

