# Supplementary material for: Prevalence, intensity and risk factors of tungiasis in Kilifi County, Kenya II: Results from a school-based observational study
Source: PLoS Negl Trop Dis. 2019 May 16;13(5):e0007326. doi: 10.1371/journal.pntd.0007326 (PMC6522002; doi:10.1371/journal.pntd.0007326)
Supplement: S1 Annex — (DOCX) [file pntd.0007326.s001.docx]

# School Informed Consent

***To be read out at a parents’ meeting***

The Ministry of Health in Kenya would like to control jiggers in all areas of the country. To start with they must find out who gets jiggers, why and where. With this information they can then design the best way to fight jiggers and stop the suffering.

In order to do this, the Wajimida Jigger Campaign is working with the University of Berlin to conduct the necessary surveys in schools and in homesteads. Your school has been selected to participate in the study. We would like to ask the head teacher some questions about the school. We will be observing things in the school compound such as whether there are any animals, if there is water available, any toilets and what the buildings and the floors are made of. We would like to look carefully at the feet of all students to observe whether they have jiggers, and how many they have. For those students with jiggers, we would also like to ask them some questions about their homes, their family and how they get to school.

The study team will include members of Wajimida Jigger Campaign, Dabaso Community Unit and a visiting PhD student from Germany, Susanne Wiese.

The study will not be taking any samples from anyone. We will only ask questions and make observations. The information we collect will be entered into a computer together with that from all of the schools that we visit. Someone with special training will analyse the information. No one from your community will ever see the answers of your child to the questions. The forms will be kept in a locked office away from your community. It is important that we have the names of infected students so that the treatment team can come back to treat them all again.

While we will not share individual information, we will come back to tell you about what we found for all of the schools together and how we will use that to plan the jigger control. We will also share these findings with other organisations and to the Kilifi County and national health management teams.

When we have finished asking questions another group will treat anyone who has jiggers and they will return to provide treatment on another day and make sure that the jiggers are all dead. If anyone is found to have any other disease we will advise you on the best way to manage it. No one will be given anything for participating in the study, except treatment of their jiggers if they have them.

Participation in the study is voluntary. If you do not want us to examine your child or to ask them any questions about themselves, you and your home, please tell the head teacher after this meeting or any time over the next one week. The head teacher will provide a list of those to be excluded from the study and will sign a consent form approving the study for the rest of the students.

**School Informed Consent**

**Consent of the Legally Authorized Representative; the Head Teacher**

It is up to you to decide whether you want the school and students to take part in this study. You understand that you are being asked to serve as the Legally Authorized Person and give permission for all students, except those on the exclusion list, to participate in this research study.

Signature of Head Teacher Date

Printed Name Head Teacher

**Statement of Person Obtaining Informed Consent (Wajimida Representative)**

I have carefully explained to the Head Teacher and the parents during a meeting what they can expect from their participation. I hereby certify that when this person signs this form, to the best of my knowledge, he/ she understands:

- What the study is about;
- What procedures will be used;
- What the potential benefits might be; and
- What the known risks might be.

I can confirm that this research subject speaks the language that was used to explain this research and is receiving an informed consent form in the appropriate language. This person is able to read the form and has been given a copy to keep. This subject does not have a medical/psychological problem that would compromise comprehension and can, therefore, give legally effective informed consent.

In addition, I confirm that all of the elements of informed consent have been presented to the subject according to the summary presented to the Pwani University IRB for review and approval.

_________________________________________________ ____________

Signature of Person Obtaining Informed Consent Date

__________________________________________________

Printed Name of Person Obtaining Informed Consent
